# Book Reviews

**Published:** 1800-03

**Authors:** 


### CRITICAL RETROSPECT

OF

### MEDICAL AND PHYSICAL LITERATURE.

[ FOREIGN AND DOMESTIC. ]

### MEDICINE AND SURGERY.

*Essays on the Venereal Disease and its concomitant Affections; Part the Second, &c.* By WILLIAM BLAIR, A. M. F. M. S. &c.

[ Continued from our last Number, p. 190. ]

In justice to the talent, assiduity, and moderation displayed by Mr. Blair, in this laborious controversy, we shall furnish those of our distant readers, who are not yet in the possession of this important essay, with the following instructive corollary:

" From the general mass of evidence before me, as well as from my own recent trials (which, though extensive and greatly varied, need not be here detailed) I venture to pronounce that permanent good may be derived from the judicious administration of the new remedies. Indeed, so much benefit, I think, will accrue from their use, as fully to recompense the labour of those who have most exerted themselves in the present controversy. By making this free concession, I do not mean to give the merit of a new discovery to Mr. SCOTT, nor to any of the modern experimenters: for acids have been long employed by other practitioners in medicine. Modern writers, however, deserve commendation for bringing these remedies forward to public notice, and attempting to define their virtues more explicitly. For my own part, I seem convinced that several particulars have been already ascertained; and that future observers will not be able to invalidate the following conclusions:

" I. Dyspeptic and debilitated venereal patients, if they be not hectical, almost certainly receive benefit from the daily use of the acids, in conjunction, or given alternately with mercury. They not only have their general strength and appetite improved, but are also enabled to bear the proper quantity of mercury much better than when it is administered alone.

" II. In erysipelatous, phagedænic, languid, fistulous, and irritable ulcers, where no venereal infection exists, and where mercury would probably do harm, the diluted acids are sometimes astonishingly efficacious, employed externally as well as internally.

" III. Old chronic pains and tumors in the bones, ligaments, and membranous parts have been alleviated by the internal use of the new remedies; especially when these symptoms arose from the mal-administration of mercury.

" IV. The nitric lotion is often serviceable in cases of excoriated ated glans or prepuce, &c. accompanied with a puriform discharge, where the degree of swelling, pain, and inflammation are inconsiderable; but the common saturnine wash appears to be equally beneficial, and has the advantage of never increasing the inflammatory symptoms.

" V. Gonorrhœa and leucorrhœa may now and then be removed by the acids, employed internally, or by injection; but they often will produce a troublesome dysuria, and are not so certain in arresting those discharges as the common means of cure.

" VI. Buboes tending to suppuration, and indurated lymphatic glans, have sometimes been dispersed by these medicines; but in this respect likewise they are inferior to other modes of treatment.

" VII. Although the result of my own experience has not encouraged me to persist in the use of M. Alyon's " oxygenated lard," several practitioners in London have composed an ointment (impregnated with nitrous acid) which is highly serviceable in herpetic, impetiginous, and itchy eruptions. In such cases, I have repeatedly seen good effects from the nitric mixture and lotion.

" VIII. Most of the local inconveniencies which arise from an incautious use of mercury, such as ulcerated cheeks, swelled tongue, spongy gums, loose teeth, fœtid breath and profuse salivation, however paradoxical it may seem, have been more speedily and effectually relieved by the internal exhibition of the acids, than by any other medical treatment hitherto employed: so that, for these purposes, I now trust to them confidently, and almost exclusively.

" IX. Under no circumstances of disease or peculiarity of constitution, has the oxygenated muriat of potash appeared to me preferable to the acids: but, on the contrary, the latter have proved much more beneficial, and less injurious to the system, than the former. This remark, perhaps, will hold equally true of the oxygen gas; respecting which, however, the evidence is at present too defective to ground any solid conclusion upon. I think also, that very little reliance can be placed in the nitric acid bath, except for some cutaneous affections.

" X. I have never derived any manifest advantage from increasing the daily quantity of the acids to more than two drachms; and in common, I find *one measured drachm* sufficient—either diluted in plain water, or qualified with syrup, opium, or ardent spirits. For a lotion or injection, I mix from twenty to sixty drops of the concentrated acid with a pint of pure water.

" XI. Of all the different acids, I have seen most benefit from the nitrous or nitric. The latter is more palatable, though not more efficacious, than the former: but in certain constitutions, none of the acids will agree; and in some cases, especially where considerable inflammation exists, it is highly improper to exhibit them. When they did not speedily improve the appetite, and afford an increase of vigour, I have seldom seen any future benefit to the general health from their continued exhibition.

" XII. Where the ' saline antisyphilitics,' as they are called, have disagreed, some of the following unpleasant consequences ensued; fued: viz. violent naufea, vomiting, flatulency, cardialgia, eroding pains in the ftomach, diarrhœa, dyfentery, obftinate conftipation, heat in the bowels, conftant itching of the fkin, miliary eruptions, univerfal tremor, frequent cold fhivering, extreme giddinefs, throbbing in the head, difordered intellect, erethifmus, irregular palpitation of the heart, intermitting and quick pulfe, dyfpnœa ardor urinæ, forcing pain of the uterus, diminifhed or fuppreffed fecretion of bile, fpitting of blood, hæmorrhage from the nofe, ophthalmia, and phlogiftic diathefis; to which may be added (in fome few examples) an injurious effect on the enamel of the teeth, inflamed lips, fwelled cheeks, deep ulcers of the tongue, and copious ptyalifm.

" XIII. I regard the chemical explanation which has generally been given of the *modus operandi* of the new remedies, and of mercury in the venereal difeafe, as entirely hypothetical. But whatever be their refpective mode of action, their fenfible effects are not ftrictly analogous to each other: for the falivation now and then arifing from the free ufe of the acids, is very different from a mercurial ptyalifm, being unaccompanied with loofnefs of the teeth, fpongy gums, or fœtid breath; and their conftitutional effects, in many particulars, feem of an oppofite nature from thofe which are experienced by a long continued courfe of mercury."

---

*Of the Imagination, as a Caufe and a Cure of the Diforders of the Body; exemplified by Fictitious Tractors. Read to the Literary and Philofophical Society of Bath. By* JOHN HAYGARTH, M. D. F. R. S. &c. &c. 8vo. pp. 43. 1s. 6d. 1799. Cadell and Davies.

The experiments recorded in this well written pamphlet are made with due philofophic circumfpection and accuracy; they fatisfactorily prove, that wooden and painted tractors, fo as to refemble thofe of Perkins, are fully as efficacious in the cure of difeafes, and even exceed in virtue the metallic ones, contrived by American ingenuity.

In the firft fection, Dr. Haygarth gives us the following hiftorical information: " A fair opportunity," fays he, " was offered to difcover whether the metallic tractors poffeffed any efficacy fuperior to the ligneous tractors, or wooden pegs.

" In the decifion of this queftion it ought to be duly confidered, that the chronic rheumatifm is a very obftinate and permanent diforder; that out of the five cafes, (being all who were fubjected to the trial) four of the patients believed themfelves immediately, and three remarkably relieved by the falfe tractors; and, that this report is founded upon the unanimous teftimony of five medical witneffes.* This evidence is not inferior to what is alleged in favour

---

* Dr. FALCONER, of Bath; Mr. NICHOLLS and Mr. PHILLOT, Surgeons to the Hofpital of that city; Mr. FARNELL, Apothecary at the fame Inftitution; and the Author of this Effay.

vour of the true tractors, especially if it be considered that the cases, which have been published, are selected from many which were unsuccessful, and passed over in silence. This success of the false tractors can only be exceeded by the exaggerated stories which, for some months past, have been reported in every company with increasing amazement and credulity." p. 5.

The author also informs us, that a pair of wooden tractors was sent to two medical friends, Sir William Watson, in London, and Dr. Moncriefe, in Bristol, requesting them to make similar experiments in both those cities. A letter from Mr. Richard Smith, an ingenious surgeon at Bristol, was the result of this communication. Fortunately for the good cause, the investigation of this important subject was undertaken by men who combine industry, candour, and sagacity, adequate to the task, with an ample share of learning. Such are the ten cases described by Mr. Smith, that no friend to medicine and humanity will read them without deriving much satisfaction.

The remarks subjoined to these interesting cases, by the author, deserve to be generally known.

"Thus, our inquiries," says Dr. Haygarth, "which were successfully instituted at the Bath General Hospital, have been amply confirmed in the Bristol Infirmary, by Mr. Smith, and other most respectable witnesses. The whole constitutes a body of evidence, which, in several points of view, affords very important instruction.

"The doctrine of Perkinism has been honoured by so many proselytes in America, England, and the continent of Europe, (even including some of the medical profession) that the question might, of itself, merit public discussion. But I should not have been disposed to publish a refutation of an error, which would probably be temporary, if the experiments above recited, had not appeared to warrant still more useful and general conclusions.

"I will not affront the good sense of the reader, by supposing that any apology can be required for the very innocent contrivance here practised, by inspiring a patient with the cheering affection of hope, and by slightly touching the skin with a piece of wood, in order to perform so important a professional duty, as to discriminate true from false remedies.

"When the event of our inquiries at the Bath Hospital was first communicated to the enthusiastic believers in the efficacy of the metallic tractors, the intelligence excited great commotions, accompanied with threats and abuse. A counter declaration was to be signed by a great number of very respectable persons; but I felt much reluctance to provoke them to record their names as dupes of a popular delusion, by which they might be exposed to ridicule through life. By a little delay, all such apprehensions appear to be effectually obviated.

"The influence of the passions upon disorders of the body, has been excellently illustrated by physicians of superior understanding; as, Sir George Baker, Dr. Falconer, and others. The experiments riments above related ſtrongly confirm, and even extend, our knowledge on this ſubject. As many of the effects may appear wonderful, they required to be ſupported by the moſt reſpectable teſtimony. And none will deny, that the witneſſes here produced are ſufficiently numerous, impartial, and intelligent." pp. 24 and 25.

